# The Association of Unfavorable Traffic Events and Cannabis Usage: A Meta-Analysis

**DOI:** 10.3389/fphar.2018.00099

**Published:** 2018-02-12

**Authors:** Sorin Hostiuc, Alin Moldoveanu, Ionuţ Negoi, Eduard Drima

**Affiliations:** ^1^Department of Legal Medicine and Bioethics, Carol Davila University of Medicine and Pharmacy, Bucharest, Romania; ^2^Faculty of Automatic Control and Computers, Polytechnic University of Bucharest, Bucharest, Romania; ^3^Department of Surgery, Carol Davila University of Medicine and Pharmacy, Bucharest, Romania; ^4^Clinical-Medical Department, Faculty of Medicine and Pharmacy, University Dunǎrea de Jos, Galaţi, Romania; ^5^Galai Psychiatry Hospital, Galaţi, Romania

**Keywords:** cannabis, driving under the influence of cannabis, death, injury, collision, inverse variance heterogeneity

## Abstract

**Background:** In the last years were published many epidemiological articles aiming to link driving under the influence of cannabis (DUIC) with the risk of various unfavorable traffic events (UTEs), with sometimes contradictory results.

**Aim:** The primary objective of this study was to analyze whether there is a significant association between DUIC and UTEs.

**Materials and Methods:** We used two meta-analytical methods to assess the statistical significance of the effect size: random-effects model and inverse variance heterogeneity model.

**Results:** Twenty-four studies were included in the meta-analysis. We obtained significant increases in the effect size for DUIC tested through blood analysis, with an odds ratio (OR) of 1.97 and a confidence interval (CI) between 1.35 and 2.87; death as an outcome, with an OR of 1.56 and a CI between 1.16 and 2.09; and case–control as the type of study, with an OR of 1.99 and a CI between 1.05 and 3.80. Publication bias was very high.

**Conclusion:** Our analysis suggests that the overall effect size for DUIC on UTEs is not statistically significant, but there are significant differences obtained through subgroup analysis. This result might be caused by either methodological flaws (which are often encountered in articles on this topic), the indiscriminate employment of the term “cannabis use,” or an actual absence of an adverse effect. When a driver is found, in traffic, with a positive reaction suggesting cannabis use, the result should be corroborated by either objective data regarding marijuana usage (like blood analyses, with clear cut-off values), or a clinical assessment of the impairment, before establishing his/her fitness to drive.

## Introduction

In the last years were published numerous epidemiological studies that tried to link driving under the influence of cannabis (DUIC) with the risk of various unfavorable traffic events (UTEs) – collision, injury, or death. Most of them had important limitations (see e.g., Gerberich et al., 2003; Laumon et al., 2005; Asbridge et al., 2014). For example, some articles did not differentiate between testing for tetrahydrocannabinol (THC) and its core metabolite – 11-Nor-9-carboxy-Δ^9^-tetrahydrocannabinol (THC-COOH) (Laumon et al., 2005). THC-COOH is formed through the hepatic oxidation of the active metabolite, after which is conjugated with glucuronide (Skopp and Pötsch, 2002), resulting in a water-soluble substance that can be easily excreted (Law et al., 1984). Unlike THC, which has a half-life of about 7 h (Bédard et al., 2007), THC-COOH can be detected in body fluids and may give a positive test for cannabis use for several days (or even weeks in heavy users), even though the active component is absent (Ashton, 2001), leading to a false belief that the person is DUIC. Additionally, in the terminal elimination phase of the metabolite, a single subject may produce consecutive specimens that could be tested positive, negative, and again positive, making it very hard to differentiate a new episode of consumption from a previous cannabis exposure (Goodwin et al., 2008). There is always some delay between UTE and the moment of collecting biological samples, which makes the simple determination of the relationship between cannabis use and collision risk very difficult.

Many studies analyzed the association between cannabis use and UTEs through “self-reporting,” a method known to underestimate the actual proportion of cannabis users (Gerberich et al., 2003; Asbridge et al., 2014), as many users tend not to report consumption of an illegal substance. Also, it is possible that a driver may have a positive result for cannabis, be involved in a car crash, but not be in an impaired driving status. Some studies evaluated the association between DUIC and UTE through epidemiological surveys, other used public datasets, culpability studies, or case–control studies. Some articles analyzed the association between previous use of cannabis and the risk of traffic events, showing an increased risk (Blows et al., 2005; Mann et al., 2007; Terry-McElrath et al., 2014) while other reached inconclusive results (Gerberich et al., 2003; Asbridge et al., 2014).

Also, three recent meta-analyses tried to summarize the effect size of DUIC (Asbridge et al., 2012; Li et al., 2012; Rogeberg and Elvik, 2016) and suggested that the risk of UTEs is increased by cannabis (Asbridge et al., 2012; Li et al., 2012; Rogeberg and Elvik, 2016) used a random-effect model to assess the effect size of cannabis use on UTEs, but they failed to provide prediction intervals (PI) for their values. Rogeberg and Elvik (2016) used a meta-regression model for estimating the publication bias, which was one of the objectives of our study.

### Aim

The primary objective of this study was to analyze whether there is a significant association between DUIC and UTEs.

Secondary objectives:

(1) To test whether DUIC is associated with an increased risk of unfavorable driving-related outcomes compared to chronic cannabis use, based on recent published literature (after 2000).
(2) To test whether publishing bias is significant in studies dealing with cannabis use in drivers.
(3) To see whether the self-reported use of cannabis during driving leads to an under-reporting of the actual cannabis use while driving.

## Materials and Methods

The study was performed by following PRISMA and MOOSE guidelines for reporting systematic reviews and meta-analyses of observational studies in epidemiology.

### Selection Criteria

Inclusion criteria: (1) observational studies, with control or comparison group, published after 2000, in which cannabis usage was associated with UTEs. We used as exclusion criteria: (1) the absence of relevant information to reconstruct the data needed for the analysis, (2) case series and studies without a control group, (4) studies not published in English, and (5) studies not published as scientific articles. Cannabis use was assessed by detection of THC in blood, metabolites in urine or saliva, or through self-report of cannabis use previous to the car crash. Chronic cannabis use was assessed through self-reports. Chronicity was taken directly from the studies, and its assessment was, therefore, author-specific.

### Search Method

We analyzed the results obtained from three databases: ISI Web of Science, Pubmed, and Scopus. For Pubmed, we used the following keywords: accidents, traffic [MeSH Terms] OR motor vehicle [MeSH Terms] OR collision [MeSH Terms] AND cannabi^∗^ OR marijuana [MeSH Terms] OR THC [MeSH Terms]. For Scopus and Web of Science, we used similar keywords, depending on the specifics of each database. We preferred not using additional restrictive criteria like article type because some other varieties (reviews, case presentations, letters to the editor) were considered as potentially adding relevant information to the meta-analysis (discussions, finding other relevant articles).

### Data Collection and Analysis

For selected studies, two reviewers extracted the data separately and included it in Excel datasheets. We summarized the following information: study, year, the total number of cases, country, type of study, type of consumption (acute/chronic), methods of detecting cannabis use, interferences with drinking alcoholic beverages, outcome, mean age and sex ratio for each group, and statistical data. We used the following types of information (in the preferred order): case–control 2 × 2 data, OR and CI, OR (ln(OR)) and SE, or RR. We transformed RR to OR by using the following formula: OR = RR(1-*p*)/(1-*p*RR), where *p* was the prevalence of cannabis use in that country, taken from the EMCDDA datasheets for Europe or CAS/CADUMS database for Canada. The agreement rate between researchers was 94%. Where we found discrepancies, the issues were analyzed by a third reviewer.

### The Risk of Bias

The risk of bias was assessed separately for each case, at a study level, and it was included in the quality assessment. We included selection bias, multiple publication biases (reason for removing two articles from the meta-analysis), and sampling bias.

### The Quality Assessment

The quality assessment included the examination of the ensuing data: (1) number of cases (including the ratio cases/controls); (2) the type of study; (3) usefulness of data (a good identification of adjusted, and non-adjusted odds ratios (ORs), clear differentiation between DUIC and previous cannabis use, differentiation of cannabis usage alone from cannabis with other drugs, blood sampling versus self-reports); (4) recruitment strategy; (5) a clear differentiation between cannabis and alcohol use in the study; (6) methods of detecting cannabis; (7) external validity; and (8) a proper assessment of the limits of the study.

Based on the elements mentioned above, we drafted a 21 points scale that we used to separate the studies into high-quality, medium-quality, and low-quality. We obtained the score for each study by dividing the obtained value by 21. We then considered as (1) high-quality studies those whose score was above mean + standard deviation; (2) medium-quality those whose score was between mean - standard deviation and mean + standard deviation; (3) low-quality those whose score was below mean - standard deviation. The scale was computed separately by two researchers. The agreement rate between researchers was 89%. All differences in evaluating the quality of a study were analyzed by a third reviewer.

### Statistical Analysis

We determined the effect size in all cases using a random-effects model computed in Microsoft Excel 2013 with MetaXL package and verified by using CMA v2 software. For each group and subgroup, we performed a forest plot. For the analysis of publication bias, depending on the type of sub-analysis, we used the following: funnel plot, Rosenthal’s fail-safe N, and Duval and Tweedy’s Trim and Fill technique. For comparison of the effect size between two groups, we used the *Z*-test method. PI and the comparison of the effect size between groups were performed by using Microsoft Excel 2013. We also computed the effect size using a novel method that was developed specifically to reduce heterogeneity and aimed to replace the random-effects model, namely the inverse variance heterogeneity (IVhet). The model was constructed using Microsoft Excel 2013 with MetaXL package. We used 95% confidence and PIs; we considered a *p*-value <0.05 to be statistically significant; and an effect size to be small at OR values of around 1.44, medium at OR values around 2.47, and large at OR values around 4.25.

## Results

### Search Synthesis

**FIGURE 1:**
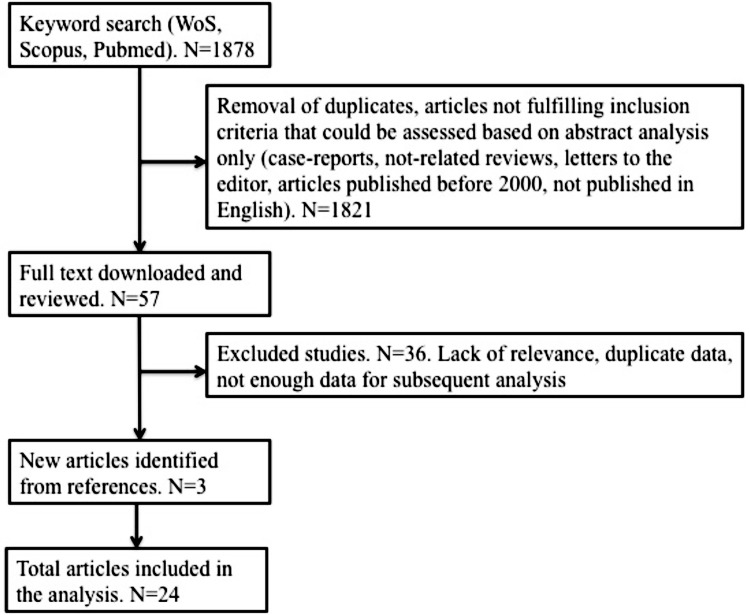
Search synthesis.

We obtained 1878 results from which, after deleting duplicate and irrelevant studies, and analyzing the type of paper and abstracts (if available), we selected 57 articles. They were downloaded and analyzed further. Scrutinizing the references of these 57 articles, we identified three more relevant papers. From the total number of 60 articles, we selected 24 for the current study, which completely respected the inclusion criteria and were encompassed in the meta-analysis. Details are presented in **Figure 1**. We only selected studies published as scientific articles, as one of the objectives of this study was to assess publication bias. We detailed the papers included in the analysis in **Table 1**. If two articles contained overlapping data, the newest article was removed from the analysis.

**Table 1:** Synthesis of the studies included in the meta-analysis.

Reference	Year	Geographical area	Sample size	Study design	Cases	Outcome	Type of data	Detection details
Asbridge et al., 2014	2014	Canada	860	Case control	Drivers involved in collisions	Injury	Blood, self-report	Active THC metabolite was measured in blood if the driver came to the emergency department within 6 h of the collision. The limit of detection was 0.2 ng/ml. Confounders: alcohol concentration, benzodiazepines, and cocaine. Self-report analyzed usual patterns of substance use over the past 6 months, including harmful use measured through the Alcohol Use Disorders Identification Test (AUDIT) and the Cannabis Use Disorders Identification Test (CUDIT)

Asbridge et al., 2005	2005	Canada	6033 (3,191/1,964/878)	Survey	Driving within 1 h after using marijuana or chronic marijuana use	Collision	Self-report	Self-reported questionnaire containing 100 items requesting information about demographics, social environment, substance use, gambling, school rules, mental health, and help-seeking

Bédard et al., 2007	2007	Canada	32,543	Case control	Drivers, with at least one potentially unsafe driving action recorded in relation to the crash	Death	Official databases	Interrogation of the FARS database (1993–2003), for drivers who had at least one potentially unsafe driving action recorder in relation to the crash versus subjects without such driving actions, who had been tested for cannabis and had a alcohol concentration of 0

Blows et al., 2005	2005	New Zeeland	1,159 (552/587)	Case control	Drivers, involved in crashes, driving within 3 h after using marijuana	Injury/death	Self-report	Telephone administered questionnaire. Case vehicles were defined as all cars involved in crashes where at least one person was hospitalized with injuries, or killed. Controls were selected through random cluster sampling. The medical records of the cases were examined, and relevant information (such as BAC) were obtained. Marijuana use was determined through two questions: usage of marijuana in the 3 h prior to the crash; for habitual use – the subjects were asked about the frequency of marijuana use in the last 12 months

Chipman et al., 2003	2003	Canada	758	Case control	Drivers with a history of cannabis use	Collisions	Self-report	Self-report

De Boni et al., 2011	2011	Brazil	609	Cross-sectional	Drivers, various groups	Injury	Self-report, saliva	Self-report. Screening of marijuana use on saliva tests analyzed through an ELISA assay, with a cut-off of 4 ng/ml THC

Fergusson et al., 2008	2008	New Zeeland	936	Retrospective cohort	Drivers, cannabis users	Collision	Self-report	Self-report, during the Christchurch Health and Development Study

Gjerde et al., 2011	2011	Norway	10,744 (204/10,540)	Case control	Drivers, fatally injured	Death	Blood	Data on blood samples submitted for forensic toxicological analysis of alcohol or drugs at the Norwegian Institute of Public Health (NIPH). Cases were excluded if the time lapse between accident and death was more than 1 day. Drug findings were confirmed and quantified using gas chromatography with mass spectroscopy detection (GC–MS) or LC–MS. The cut-off values for THC was 0.6 ng/ml in blood

Hels et al., 2013	2013	Belgium, Finland, Denmark, Italy, Lithuania, Netherlands	18,322	Case control	Severely injured drivers	Injury	Saliva/blood	Oral fluid and/or blood samples were collected at the road side (controls) and blood was sampled in the hospitals (cases), using country-dependent methods

Kuypers et al., 2012	2012	Belgium	3095 (337/2758)	Case control	Hospitalized drivers	Injury	Blood	Blood analyses performed by validated GC–MS. Cut-off for THC – 1 ng/ml

Laumon et al., 2005	2005	France	9772 (6766/3006)	Case control	Fatally injured drivers	Death	Blood	Blood analyses performed by GC–MS. Cut-off for THC – 1 ng/ml

Li et al., 2013	2013	United States	8456 (737/7719)	Case control	Fatally injured drivers	Death	Blood/urine/saliva	Drug tests for cases were performed using blood and/or urine specimens through liquid/gas chromatography, mass spectrometry, and radioimmunoassay technique. For controls were used oral liquid samples, which were first screened for drugs through the enzyme-linked immunosorbent assay and then analyzed for confirmation through chromatography and spectrometry techniques

Longo et al., 2000	2000	Australia	1975 (1038/937)	Case control	Culpable drivers	Injury	Blood	Blood samples tested for THC and THC-COOH. THC concentration was divided into drug free, 1 ng or less, 1.1–2 ng, and 2.1 or more ng/ml

Mann et al., 2007	2007	Canada	2676	Survey	Cannabis users, driving within 1 h after using marijuana	Collision	Self-report	Self-reported, through telephone-based questionnaires

Mir et al., 2012	2012	Pakistan	857	Survey	Drivers, consuming cannabis	Collision	Self-report	Self-reported through interview-based questionnaires

Movig et al., 2004	2004	Netherlands	926 (110/816)	Case–control	Injured drivers	injury	Urine/blood	Urine samples were screened with an enzyme multiplied immunoassay technique and confirmed with GC–MS. Drug screening in serum was done with enzyme immunoassay and confirmation was done with GC–MS

Mura et al., 2003	2003	France	1800 (900/900)	Case–control	Injured drivers	Injury	Blood	Blood samples, taken with an average time of 1.8 ± 0.9 h after a car crash. Subjects were screened for THC, 11-OH-THC, and THC-COOH with GC–MS/HPLC, cut-off for THC: 1 ng/ml

Pulido et al., 2011	2011	Spain	503	Case–control	Regular cocaine users recruited from non-treatment centers	Injury	Self-report	Self-reported usage, 60 and 120 min prior to a car crash

Romano et al., 2014	2014	United States	5190 (1766/3424)	Case–control	Injured drivers	Injury	Official databases	FARS database, 2007–2008

Terry-McElrath et al., 2014	2014	United States	72,053	Retrospective cohort	Cannabis users	Collision	Self-report	Questions about self-use of marijuana in the last 12 months

Woratanarat et al., 2009	2009	Thailand	1049 (200/849)	Case–control	Injured drivers	Injury	Urine	Urine samples, tested through GC–MS, cut-off 50 ng/ml

Gerberich et al., 2003	2003	United States	64,657 (188/64469)	Retrospective cohort	Members of a medical insurance program aged 15–49 years, variable patterns of marijuana use	Collision	Self-report	Self-report of marijuana use, as classified in never use, experimental use, former use, and current use

Gmel et al., 2009	2009	Switzerland	486	Case–control	Injured drivers	Injury	Self-report, blood	Self-reported use, 6 h before a car crash. Subjects were screened for THC, 11-OH-THC, and THC-COOH. Cut-off for THC: 0.5 ng/ml

Jones et al., 2005	2005	Australia	320	Retrospective cohort	Cannabis users involved in crashes, dependent	Collision	Self-report	Self-reported use of cannabis


### Quality Assessment

**FIGURE 2:**
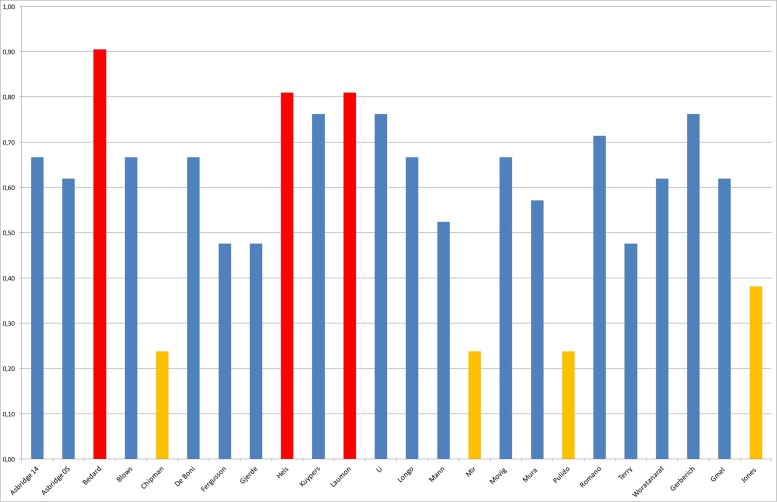
Quality assessment.

The final mean score was 0.62 and the standard deviation was 0.19. The distribution of the quality scores is shown in **Figure 2**.

### Driving under the Influence of Cannabis Unadjusted

**FIGURE 3:**
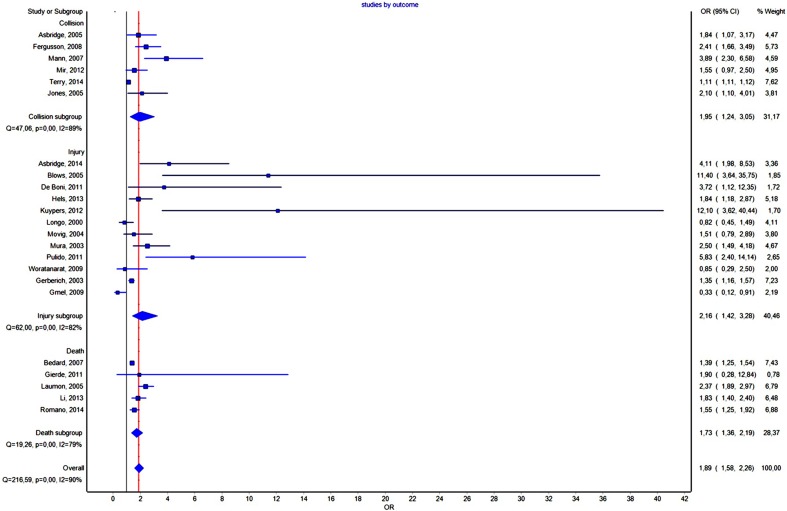
Forrest plot. Studies grouped by outcome (RE Model).

Twenty-three studies contained unadjusted data about DUIC (**Figure 3**). The effect size was mild, with OR = 1.889, CI = (1.580–2.258) and a PI between 0.92 and 3.84, not statistically significant (PI overlapped the value 1).

**FIGURE 4:**
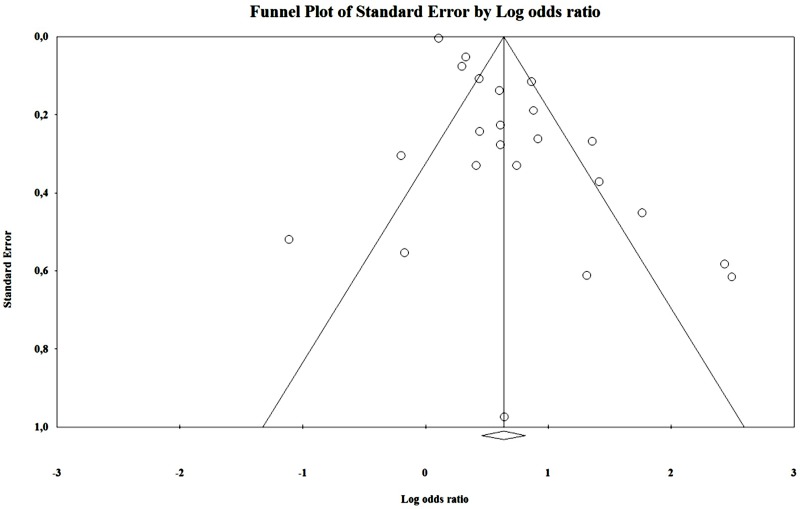
Funnel plot – unadjusted.

By analyzing the funnel plot (**Figure 4**) of this distribution, we saw an increased asymmetry number of cases outside the funnel, implying a potential publication bias. The Rosenthal fail-safe N gave a *Z*-value of 19.01 (*p* < 0.001), being needed 2143 missing studies to bring the *p*-value over alpha (1.96). The Duval and Tweedie’s Trim and Fill method adjusted the OR to 1.21 (1.01–1.44). The effect size, as computed using the IVhet method, was 1.12 (0.59–2.12).

### Driving under the Influence of Cannabis–Blood Analysis

**FIGURE 5:**
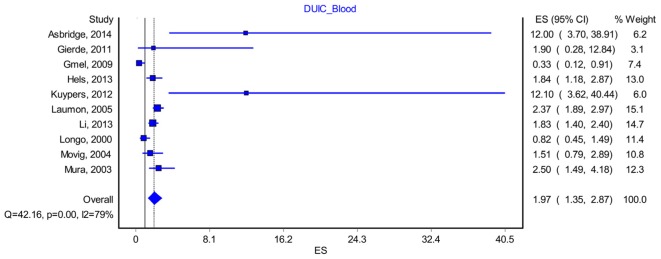
Forrest plot. Studies that estimated DUIC through blood analysis (RE Model).

Ten studies included data that allowed us to reconstruct a proper methodological blood analysis of the samples taken from drivers (Longo et al., 2000; Movig et al., 2004; Laumon et al., 2005; Mura et al., 2006; Gmel et al., 2009; Gjerde et al., 2011; Kuypers et al., 2012; Hels et al., 2013; Li et al., 2013; Asbridge et al., 2014). By including them in the analysis, we found a modest increase in the OR to 1.97, CI D (1.35–2.87), with a PI of 0.59–6.49 (**Figure 5**). The effect size difference between the values obtained for “DUIC-unadjusted” and “DUIC–blood analysis” was not statistically significant (*Z*_diff_ = -0.19, *p* = 0.84). The Rosenthal fail-safe N had a *Z*-score of 3.18 (*p* < 0.001), suggesting that there should be added 171 missing studies to bring the *p*-value over alpha (1.96). The Duval and Tweedie’s Trim and Fill method did not adjust the OR (no studies were trimmed). The effect size, as computed using the IVhet method, was 2.01 (1.23–3.29).

### Chronic Cannabis Use

**FIGURE 6:**
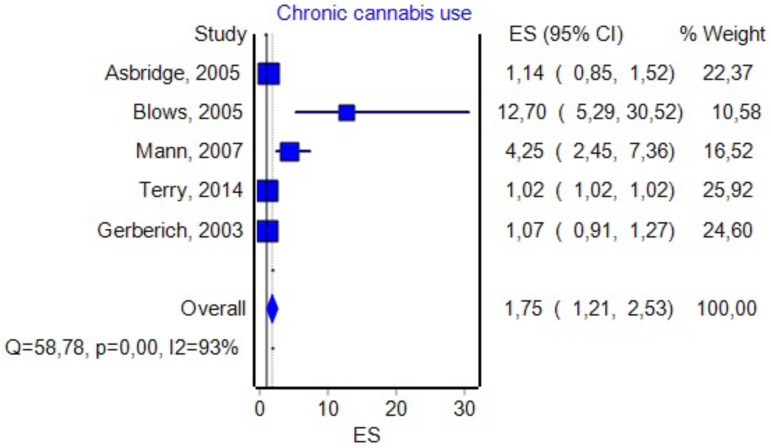
Forrest plot. Studies that estimated chronic cannabis use in drivers (RE Model).

Five studies had data about the effect of chronic marijuana use in relation with UTEs (see **Figure 6** for details). By including them in the analysis, we found an OR value that was similar to the one associated with DUIC 1.75, a CI between 1.21 and 2.53, and a PI between 0.46 and 6.6. The effect size difference between the values obtained for DUIC and chronic use was not significant (*Z*_diff_ = -0.36, *p* = 0.71). Similarly, the effect size difference between the values obtained for DUIC–blood analysis and chronic cannabis use was not statistically significant (*Z*_diff_ = -0.99, *p* = 0.32). The Duval and Tweedie’s Trim and Fill method decreased the OR to 1.02 (0.71–1.47). The effect size, as computed using the IVhet method, was 1.02 (0.49–2.11).

### DUIC through Self-Reports

**FIGURE 7:**
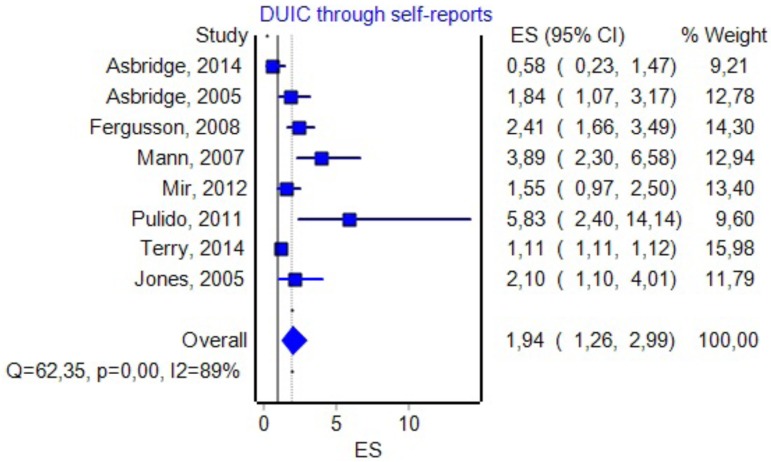
Forrest plot. Studies that estimated DUIC through self-reports (RE Model).

Eight studies included information about the effect size of DUIC, as presented through self-reports and UTEs (see **Figure 7** for details). By including them in the analysis, we obtained an OR of 1.94 (1.26–2.99) and a PI between 0.45 and 8.34. The effect size difference between DUIC–blood analyses and DUIC through self-reports was not statistically significant (*Z*_diff_ = -0.05, *p* = 0.95). The Rosenthal fail-safe N had a *Z*-score of 15.87 (*p* < 0.001), suggesting that there should be added 518 missing studies to bring the *p*-value over alpha (1.96). The Duval and Tweedie’s Trim and Fill method decreased the OR to 1.22 (0.81–1.82). The effect size, as computed using the IVhet method, was 1.12 (0.38–3.30).

### DUIC with THC Blood Levels Over 0.5 ng/ml

Three studies contained data about the effect size of DUIC with THC blood levels above 0.5 ng/ml on UTEs (Laumon et al., 2005; Gmel et al., 2009; Kuypers et al., 2012). By including them in the analysis, we obtained an OR of 2.085 (0.35–12.43) and a PI between 0.0000001 and 6107085547. The effect size, as computed using the IVhet method, was 2.28 (0.22–23.82).

### Effect Size of the DUIC Depending on the Outcome

The forest plot of the subgroup analysis is presented in **Figure 3**. A comparison of the other statistical parameters is presented in **Table 2**. For collision or injury as outcomes, the effect was not statistically significant. For death, the effect size was statistically significant using the IVhet method (1.56, CI = 1.16–2.09).

**Table 2:** Effect sizes depending on the outcome (italics – statistically significant increase in the effect size).

Outcome	Random-effect model			Trim and Fill		IVhet	
			
	OR	CI	PI	Adjusted OR	CI	OR	CI
Collision	1.95	1.24–3.05	0.41–9.20	1.22	0.82–1.81	1.12	0.41–3.03
Injury	2.16	1.41–3.28	0.50–9.3	1.47	0.94–2.29	1.54	0.66–3.59
Death	1.73	1.36–2.19	0.77–3.84	*1.43*	*1.12*–*1.83*	*1.56*	*1.16*–*2.09*


### Effect Size of the DUIC Based on the Type of Study

The comparison of effect size based on the type of study is presented in **Table 3**. We assessed retrospective, cross-survey, and survey studies together due to their low number. Neither had statistically significant effect sizes as obtained using the random-effects model separately. From **Table 3** we see that case–control studies have a significantly increased effect size using IVHet (IVHet = 1.99, CI = 1.05–3.80).

**Table 3:** Effect sizes depending on the type of study (italics – statistically significant increase in the effect size).

Type of study	Random-effect model			Trim and Fill		IVhet	
			
	OR	CI	PI	Adjusted OR	CI	OR	CI
Case–control	1.951	1.51–2.51	0.82–4.63	*1.58*	*1.20–2.07*	*1.99*	*1.05–3.80*
Other types	1.81	1.38–2.39	0.78–4.21	1.16	0.91–1.51	1.13	0.39–3.27


### Effect Size Considering Adjustments Made by Authors to the OR

**FIGURE 8:**
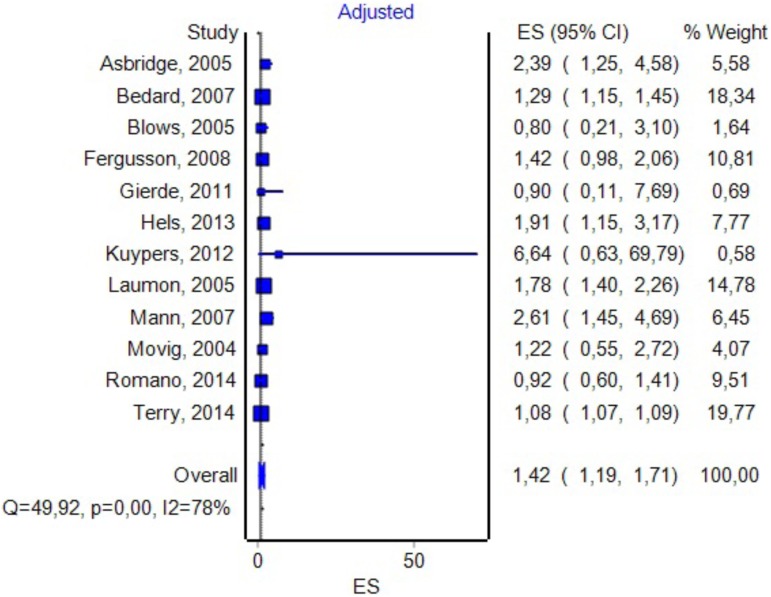
Forrest plot. Studies in which the authors provided additional adjustments to the model (RE Model).

In 12 studies, the authors adjusted the OR value for various confounding variables (see **Figure 8** for details). By including them in the analysis, we found an OR value of 1.42 with a CI between 1.19 and 1.71 and a PI between 0.85 and 3.6. The Duval and Tweedie’s Trim and Fill method decreased the OR to 1.19 (0.99–1.42). The effect size, as computed using the IVHet method, was 1.09 (0.73–1.62).

## Discussion

The association between DUIC and the risk of road traffic events was intensely studied before (Williams et al., 1985; Ramaekers et al., 2004; Mura et al., 2006; Turner, 2007; Romano and Voas, 2011; Pickett et al., 2012; Poulsen et al., 2012; Li et al., 2013; Rossheim et al., 2014; Terry-McElrath et al., 2014; Urfer et al., 2014). There are several methods of assessing this association – from experimental studies aimed to measure the influence of THC on driving performance, to epidemiological, culpability, or case–control studies. Although each add pieces of information, a definite answer is difficult to obtain, as these studies often had conflicting results and the research methodology was regularly prone to biases. Due to these reasons, a series of systematic reviews (e.g., Ramaekers et al., 2004) or meta-analysis (Asbridge et al., 2012; Li et al., 2012) were recently published, which strongly suggested an association between DUIC and UTEs.

When reporting a meta-analysis carried out using the random-effects model, the researchers usually present the summary effect size and its CI. These values allow us to estimate the mean effect size and precision but not the distribution of the true effects around the summary effect (Borenstein, 2009). Therefore, when exhibiting the result of a random-effect meta-analysis, we should detail three pieces of information: mean effect size, CI, and PI. The PI shows the distribution of the true effect sizes around the mean. Both meta-analyses (Asbridge et al., 2012; Li et al., 2012), as discussed above, used a random-effect model and obtained significant effect sizes. However, neither reported PIs for their analyses. By including the PI in the description of the results, we failed to obtain a significant association between DUIC or other computed parameters and UTEs, even if the OR and CI of our study were highly similar to theirs. Moreover, when analyzing various subgroups, we could not find statistical associations.

We could imagine three possible causes for the results obtained in our study: (1) the absence of any correlation between DUIC and UTEs; (2) a very high heterogeneity of the studies included in the analysis, which led to a very high dispersion of the true effect size; and (3) a lack of sensitivity of the random-effects model for our analysis. Riley et al. (2011) argued that using PIs is appropriate when the studies included in the meta-analysis have a low risk of bias, which was not the case in our study (see *I*^2^ values in **Figures 3**, **5**–**8**).

The heterogeneity of the results is caused both by the different methodologies used by various studies and by the technical difficulties in actually assessing impairment of driving associated with cannabis use (Fischer et al., 2006; Gorun et al., 2010; Hartman et al., 2016b). For example, it is known that the concentration in blood of THC decreases rapidly after use, but the clinical effects take longer to dissipate (Hartman et al., 2016a). This, associated with the fact that sampling is done sometimes even hours after the event, may significantly alter the results. The metabolism of cannabinoids is highly variable in different subjects; therefore, at the same concentration, a person may be under the influence, while another may have normal driving ability (Hartman et al., 2016a). There is no standardized cut-off for blood THC from which a person is considered under the influence. For more details, see National Academies of Sciences and Medicine (2017, Chapter 15).

To clarify which hypothesis is more plausible, we reanalyzed the studies, by using a newly developed approach to meta-analyses namely the IVHet model, which was developed specifically to decrease heterogeneity and increase the overall performance of the meta-analysis. Succinctly, IVHet has as key benefits over the random-effect model the lack of penalization for larger trials, a more conservative CI, and the fact that it exhibits a lesser true variance irrespective of the degree of heterogeneity (Doi et al., 2015). By using this approach, we identified a lack of significance when including in the final analysis studies that couldn’t quantify the actual DUIC using objective methods (blood tests). When DUIC was properly established using blood tests, we saw an OR suggesting a small-to-medium effect size (by also considering the CI). Case–control studies showed a significantly increased effect size; most likely the primary cause is a more frequent usage of a blood test to detect THC compared to other study designs. Also, case–control tests may misrepresent the actual situation in the population from which the cases/controls were obtained, by inadvertently pre-selecting subgroups from the population of drivers, which are more prone to risky behaviors. For example, it is possible that the control group was matched for age and sex with the case group (Mura et al., 2003), not taking into account the fact that men and younger persons tend to use cannabis more frequently. Alternatively, it is possible that the design included temporal patterns of obtaining controls (Asbridge et al., 2014), not considering that cannabis use occurs more at night or weekends (Laumon et al., 2005; Kuypers et al., 2012), when the risk of UTE is higher. Alternatively, the controls were matched geographically (Woratanarat et al., 2009), not taking into account that usually urban areas have a higher overall usage of cannabis compared to the countryside (Drummer et al., 2003; Li et al., 2012), and subsequently there are differences in the collision risk (Khorashadi et al., 2005; Zwerling et al., 2005). Some studies specifically scrutinized for this issue and adjusted the OR for confounding variables; by using this approach, they obtained inconclusive results even if they initially hinted an increased risk for cannabis users. For example, Blows et al. (2005) showed a decrease in OR from 11.4 (3.63–35.75) to 0.8 (0.2–3.1) after adjusting for confounders. By using adjusted ORs in a separate inquiry, we obtained results that were highly similar with those obtained through the analysis of unadjusted ORs.

A similar reasoning could be applied to the association between cannabis use and death. All studies that included death as the outcome were case–controls. Moreover, for the cases group, intoxication status was assessed using blood analysis; the cannabis use in control subjects was evaluated using various methods including saliva and/or urine (Li et al., 2013). Case–control studies in this field are known to have a high selection bias, caused by the fact that the two groups may be from different time periods or geographical regions, or they might include different cannabis-detection cutoffs (Hartman and Huestis, 2013).

Studies performed using other designs like retrospective cohorts or surveys often suffer from the same methodological flaws, especially preselecting participants who are more likely to use marijuana. They also rely heavily on self-reported cannabis use, that is known to under-represent the actual consumption and subsequently DUIC (Asbridge et al., 2014). Therefore, a methodology combining both types of studies would most likely obtain results that could reveal closer estimates of the true effect size of DUIC on UTEs.

We assessed publication bias both qualitatively (funnel plot) and quantitatively (Rosenthal’s Fail-Safe N and Duval and Tweety’s Trim and Fill methods). The latter were particularly useful as they allowed us to estimate the effect size after adjusting for publication bias. By analyzing the results obtained through the Trim and Fill method applied to the results of the random-effect model, we saw a sharp decrease in the effect size in most instances (except chronic cannabis use, that was usually used as a comparison/control group to acute exposure in the analyzed studies). Moreover, the results were, in most part, very close to those obtained using IVHet, suggesting that a significant proportion of the heterogeneity encountered by the random-effect model was caused by publication bias favoring studies that showed a positive association between DUIC and UTEs [see Doi and Thalib (2008) for further details].

Our analysis suggested that the effect size for unadjusted DUIC on UTEs was not statistically significant. This result might be caused by (1) methodological flaws, which are often encountered in articles on this topic [for a detailed analysis see Hartman and Huestis (2013)]; (2) the indiscriminate use of the term cannabis use (which in our study included a wide array of studies, including some in which the cut-off value for this substance was below the one known to cause a significant clinical impairment); and (3) or a true absence of a negative effect of DUIC on UTEs. Simply identifying cannabis use in a driver is not enough to justify the assumption of an increased risk for UTEs. When such a result is obtained, it should be corroborated with either quantitative data regarding cannabis use, or a clinical assessment of the driver, before establishing his fitness to drive.

## Limitations

Many limitations were already presented in the section “Discussion,” as they were intrinsically linked with the discussion of the results. Additionally, there were a few other limitation, that will be presented here. Most studies included in the meta-analysis failed to provide detailed descriptive data. For example, many didn’t present mean age for the case and control groups, making it impossible to perform a meta-regression, needed to test the degree heterogeneity explained the age of the participants. Given that there are different types of outcomes (injury, death, collision), the obtained data from the pooled studies might be inconsistent – for example, some of the injury reports have long delays from the crash to sampling limiting the detectability of THC in blood. We used a 0.5-ng/ml value as a cut-off for some analyses as this value was identified in a large enough number of articles, although a higher limit (2 or even 5 ng/ml) might have been more appropriate to test severe impairment due to marijuana abuse. A positive test for cannabis (i.e., blood) does not necessarily imply that drivers were impaired, as THC/metabolites might be detected in blood a long time after impairment, especially in chronic cannabis users, which could also induce an important bias in the analysis of the results. The unreliability of the self-reported studies cannot be properly tested. The literature of cannabis and its effects on driving ability is extremely difficult to analyze due to confounding generated by the measuring and interpreting THC, not only experimenter bias.

## Conclusion

Our analysis suggests that the overall effect size for DUIC on UTEs is not statistically significant, but there are significant differences obtained through subgroup analysis. This result might be caused by either methodological flaws (which are often encountered in articles on this topic), the indiscriminate employment of the term “cannabis use,” or an actual absence of an adverse effect. When a driver is found, in traffic, with a positive reaction suggesting cannabis use, the result should be corroborated by either objective data regarding marijuana usage (like blood analyses, with clear cut-off values), or a clinical assessment of the impairment, before establishing his/her fitness to drive.

## Author Contributions

SH – designed the study, involved in the statistical analysis, drafted the first version of the manuscript, and accepted the final version. IN – involved in the research of the studies, statistical analysis, reviewed the manuscript, and approved the final version. AM – involved in statistical analyses, proofreading the manuscript, and approving the final version. ED – involved in the research of the studies, statistical analysis, proofreading the manuscript, and accepted the final version of the manuscript.

## Conflict of Interest Statement

The authors declare that the research was conducted in the absence of any commercial or financial relationships that could be construed as a potential conflict of interest. The reviewer EO and handling Editor declared their shared affiliation.
