# Corrigendum: The Association of Unfavorable Traffic Events and Cannabis Usage: A Meta-Analysis

**DOI:** 10.3389/fphar.2018.00564

**Published:** 2018-05-29

**Authors:** Sorin Hostiuc, Alin Moldoveanu, Ionuţ Negoi, Eduard Drima

**Affiliations:** ^1^Department of Legal Medicine and Bioethics, Carol Davila University of Medicine and Pharmacy, Bucharest, Romania; ^2^Faculty of Automatic Control and Computers, Polytechnic University of Bucharest, Bucharest, Romania; ^3^Department of Surgery, Carol Davila University of Medicine and Pharmacy, Bucharest, Romania; ^4^Clinical-Medical Department, Faculty of Medicine and Pharmacy, University Dunǎrea de Jos, Galați,, Romania; ^5^Galati, Psychiatry Hospital, Galați,, Romania

**Keywords:** cannabis, driving under the influence of cannabis, death, injury, collision, inverse variance heterogeneity

1. In the results section, in the subchapter “Driving under the influence of Cannabis-Blood Analysis,” there was an error in the submitted manuscript. The error consisted in the presence of an additional reference in the studies included in the analysis, and the lack of two references from the studies included in the analysis. The overall results are very similar and do not change the scientific conclusions of the article in any way. The correct text for this subchapter, including all corrections (in **bold**), is:

“**Ten** studies included data that allowed us to reconstruct a proper methodological blood analysis of the samples taken from drivers (**Longo et al.**, **2000**; Movig et al., 2004; Laumon et al., 2005; Mura et al., 2006; Gmel et al., 2009; Gjerde et al., 2011; Kuypers et al., 2012; Hels et al., 2013; **Li et al.**, **2013**; Asbridge et al., 2014). By including them in the analysis, we found a modest increase in the OR to **1.97**, CI = (**1.35–2.87**), with a PI of 0.**5**9–**6.49** (Figure 5). The effect size difference between the values obtained for “DUIC-unadjusted” and “DUIC–blood analysis” was not statistically significant (*Z*_diff_ = −0.**19**, *p* = 0.**84**). The Rosenthal fail-safe N had a *Z*-score **3.18** (*p* < 0.001), suggesting that there should be added 171 missing studies to bring the *p*-value over alpha (1.96). The Duval and Tweedie's Trim and Fill method did not adjust the OR (no studies were trimmed). The effect size, as computed using the IVhet method, was 2.**01** (1.23–3.29).”

2. Additionally, due to the above-mentioned error, a few other small changes were made, namely:
  a. In the Abstract, Results, line 3, instead of “ with an odds ratio (OR) of 2.27 and a confidence interval (CI) between 1.36 and 3.80” should read: “with an odds ratio (OR) of 1.97 and a confidence interval (CI) between 1.35 and 2.87”
  b. In the Results chapter, the subchapter “DUIC through self-reports”, instead of “The effect size difference between DUIC–blood analyses and DUIC through self-reports was not statistically significant (***Z***_diff_ = −0.58, ***p*** = 0.56)” should read: “The effect size difference between DUIC–blood analyses and DUIC through self-reports was not statistically significant (***Z***_diff_ = −0.05, ***p*** = 0.95)”
3. Figure 5 from the article is not correct, it is a duplicate of Figure 8. The original of Figure 5 was inserted in the final version of the manuscript sent to reviewers. Attached is the correct figure, with the changes made to point 1.
4. Additionally, some readers were unclear why were some articles removed from our analysis. We have specified the fact that two articles were removed due to multiple publication bias in the materials and methods section. However, to be clearer, we would like to add another sentence in the Results section/Search synthesis, namely: “If two articles contained overlapping data, the newest article was removed from the analysis.”
5. In the Materials and Methods chapter, subchapter The Risk of Bias, line 4, instead of “and selection bias” should read “sampling bias.”

**Figure 5:**
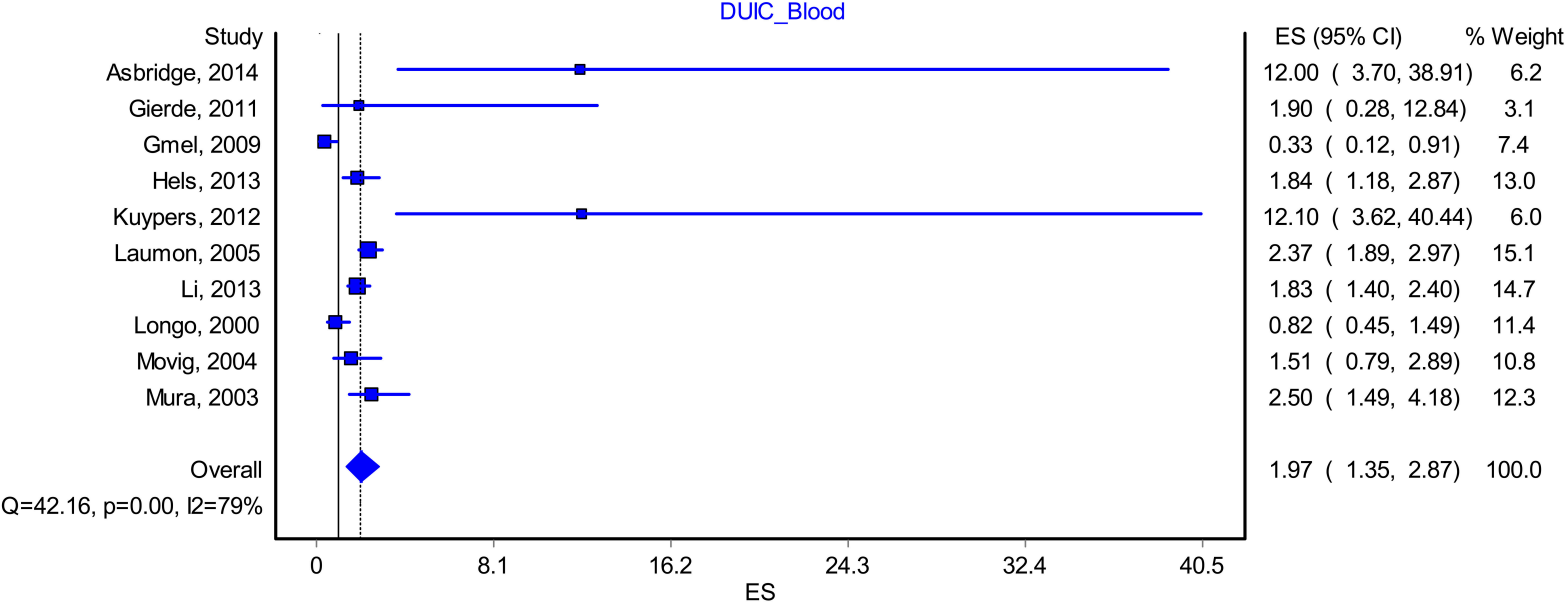
Forrest plot. Studies that estimated DUIC through blood analysis (RE Model).

The authors apologize for these mistakes. These errors does not change the scientific conclusions of the article in any way.

The original article has been updated.

## Conflict of interest statement

The authors declare that the research was conducted in the absence of any commercial or financial relationships that could be construed as a potential conflict of interest.
